# A turning point in the bacterial nanocellulose production employing low doses of gamma radiation

**DOI:** 10.1038/s41598-022-11010-4

**Published:** 2022-04-29

**Authors:** Ola E. A. Al-Hagar, Deyaa Abol-Fotouh

**Affiliations:** 1 Plant Research Department, Nuclear Research Center, Egyptian Atomic Energy Authority, Cairo, 13759 Egypt; 2 Department of Electronic Materials Researches, Advanced Technology and New Materials Research Institute (ATNMRI), City of Scientific Research and Technological Applications (SRTA-City), New Borg El-Arab City, Alexandria, 21934 Egypt

**Keywords:** Structural properties, Biopolymers

## Abstract

In the recent years, huge efforts have been conducted to conceive a cost-effective production process of the bacterial nanocellulose (BNC), thanks to its marvelous properties and broadening applications. Herein, we unveiled the impact of gamma irradiation on the BNC yield by a novel bacterial strain *Komagataeibacter hansenii* KO28 which was exposed to different irradiation doses via a designed scheme, where the productivity and the structural properties of the BNC were inspected. After incubation for 240 h, the highest BNC yield was perceived from the culture treated twice with 0.5 kGy, recording about 475% higher than the control culture. Furthermore, almost 92% of its BNC yield emerged in the first six days. The physicochemical characteristics of the BNCs were investigated adopting scanning electron microscope (SEM), thermogravimetric analysis (TGA), X-ray diffraction (XRD), and Fourier transform infrared (FTIR). Additionally, the water holding capacity, water release rate, surface area (BET), and mechanical properties were configured for the BNC generated from the control and the irradiated cultures. As a whole, there were no significant variations in the properties of the BNC produced by the irradiated cultures versus the control, proposing the strain irradiation as a valuable, facile, and cheap route to augment the BNC yield.

## Introduction

Bacterial nanocellulose (BNC) has grabbed the interest of academic and industrial researchers in the last decade because of its remarkable physical features, which include high tensile qualities, crystallinity, thermal stability, and biocompatibility^1,2^. Despite the fact that the BNC has the same chemical structure of the plant cellulose (C_6_H_10_O_5_)_n_, BNC is produced in a 3D network of nanofibers, naturally pure and free from hemicellulose or lignin, and exhibits much higher merits of surface area, water holding capacity, and polymerization^3,4^. The unique BNC’s features allowed it to be used in a wide range of applications, including biomedical and pharmaceutical fields, electro-conductive composites, the food industry, waste-water treatment, food packaging, textiles, and even the construction arena^2,5,6^.

Several genera were reported to synthesize BNC, including *Komagataeibacter, Agrobacterium, Aerobacterium, Rhizobium, Salmonella, Sarcina, Azotobacter,* and *Achromobacter,* etc., though the production yield varies greatly^7^. However, representatives of the genus *Komagataeibacter* (previously known as *Acetobacter*) were recognized to be the most efficient BNC producers, with *Komagataeibacter xylinus* serving as the role model bacterial strain for BNC production^8^. Despite the rising importance of BNC around the world, the production process still encounters a number of challenges, including long propagation periods, low production yields, and limited cellulose layer thickness, which are all key roadblocks to BNC mass production and application^9^.

Research groups have put in a lot of work to enhance the BNC yield, whether it’s the medium composition, the production conditions, or the strain efficiency. The medium accounts for over 30% of total production costs, with recent developments focusing on experimenting with cheaper carbon and nitrogen sources such as agro-industrial wastes, food remnants, paper industry effluents, and textile wastes^1,10^. Another strategy for increasing BNC yield is to optimize the production parameters, which involved testing a variety of bioreactors to find the best production conditions for the employed bacteria, either in a static or agitated state^6^.

Alternatively, selecting a high-efficiency bacterial strain for BNC manufacturing is a critical stage in the BNC mass-production process. With the same production circumstances, the used strain can be genetically tweaked to produce significantly more BNC^8^.

The bacteria’s genetic modification can be achieved either by genetic recombination, or by mutation via an external chemical or physical stimulus (termed mutagen). In order to boost the potentialities of wild-type strains, genetic recombination involves introducing genetic components into them.

Attempts at genetic recombination to improve BNC production or bestow it with certain characteristics have continued. Kuo et al. improved BNC production by around 40 and 230% in static and agitated cultures of the *Gluconacetobacter xylinus* strain, respectively, by homologous replacement of the membrane bound glucose dehydrogenase (GDH) gene with a deficient one. The GDH system was discovered to be responsible for the wasteful conversion of medium glucose units to gluconic acid at the start of the incubation period^11^. Jang and his coworkers reported that they attained the best improvement in the BNC yield by introducing *pgi* overexpression gene heterologously to the strain *Komagataeibacter xylinus* 2325, that recording about 3.15 g/L and represents 115% higher than the wild-type strain (1.46 g/L)^12^.

Another example was the genetic alteration of the *Gluconacetobacter xylinus* strain to synthesize BNC and curdlan concurrently in situ as a bio-based nanocomposite^13^. By designing the bacteria *Gluconacetobacter xylinum* to naturally add N-acetylglucosamine residues on the cellulose nanofibers, Yadav et al. altered the properties of the BNC by lowering crystallinity and raising biodegradability^14^. However, this method needs a deep grasp of the driving mechanisms of bacteria, as well as the structure and control of their genes. Obtaining a thorough understanding of these aspects is extremely rare, difficult, and costly. Furthermore, many countries, particularly in the food industry, have limits on recombinant strains^15^. Furthermore, due to the intricate regulatory process in which each gene may express a protein with various roles, genetic change may not necessarily have a positive impact on the yield or properties of the generated BNC^8^.

Gamma ray have piqued extensive attention of the biotechnologists because of its significance in genetic modification for either increased product yield or improved properties. Although it was designated as a ubiquitous sterilization agent, the treatment of live cells by low doses of gamma irradiation was reported to attain interesting mutations. Moreover, employing gamma ray irradiation is cheap and conceivable by most research disciplines^16–20^.

This work aimed to explore the implications of several doses of gamma irradiation on the BNC productivity of the strain *Komagataeibacter hansenii* KO28, as well as the physical and chemical properties of the synthesized BNCs of the irradiated cultures comparing to the control product. To the best of our knowledge, this is the first study that has explored whether the gamma ray-assisted mutagenesis of the BNC-producing bacteria can be a promising approach to optimize the BNC production or not.

## Results and discussion

The global concerns about the rising environmental hazards have increased year after year. Conceiving sustainable and green materials to substitute the non-eco-friendly synthetic polymers and the derivatives of the fossil oil has become a cosmic priority^21^. Cellulose is the most abundant natural polymer, where it comprises almost 30% of the worldwide plant matters. The universal cellulose production -mainly from plant source- estimated to be 10^10^–10^11^ tons annually, and about 6 × 10^9^ tones has its way to the paper, textile, materials and chemical industries^22^.

Bacterial nanocellulose (BNC) represents a revolutionary shift to more sustainable processes, where it can be produced utilizing wide ranges of wastes and with fair effluents; constituting a high value-added and green product. Moreover, BNC is a cellulose in a fully pure form; there is no effort-, money-, or energy-wasting extraction methods are demanded, contrary to the plant cellulose^23^.

### Identification BNC-producing strain

In the present study, the bacterial strain *Komagataeibacter hansenii* KO28 was employed to produce BNC. The strain isolated and identified through morphological and physiological examinations, in addition to the partial sequencing of the 16 s rRNA gene ^24^. More data about isolation and identification protocols can be found in the methodology part and supplementary information.

### Gamma irradiation scheme

**Figure 1:**
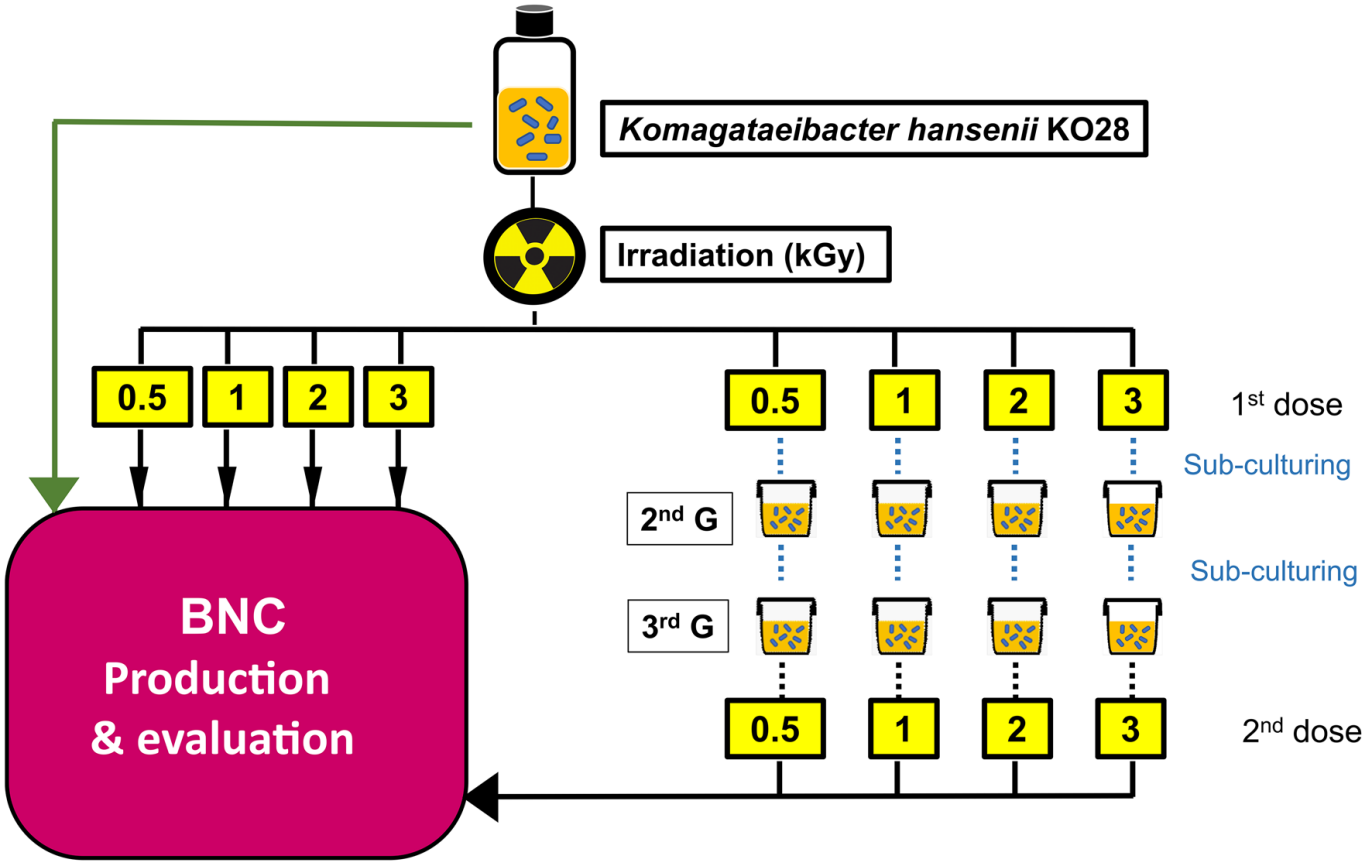
Schematic diagram explains the gamma irradiation plan adopted to induce mutation to the strain *Komagataeibacter hansenii* KO28. The irradiated cultures were examined for their BNC yield versus the control (non-irradiated) culture, while the BNC products were compared for their chemical and physical properties to inspect the irradiation influence.

Figure 1 depicts the irradiation scheme we implemented to induce genetic mutation to the strain *Komagataeibacter hansenii* KO28. Briefly, the plan comprised treating the strain cultures with gamma ray viz. 0.5, 1, 2, 3 kGy with singular and dual doses in parallel, prior the BNC production was conducted from the irradiated cultures versus the control one (non-irradiated) on Hestrin-Schramm (HS) medium for 10 days to inspect the impact of irradiation doses on the BNC productivity.

It seemed that the gamma irradiation with doses ≥ 2 kGy (singular or dual) were either not supportive for the BNC production or even lethal for the culture cells; where no BNC was observed in the cultivation vessels.

**Figure 2:**
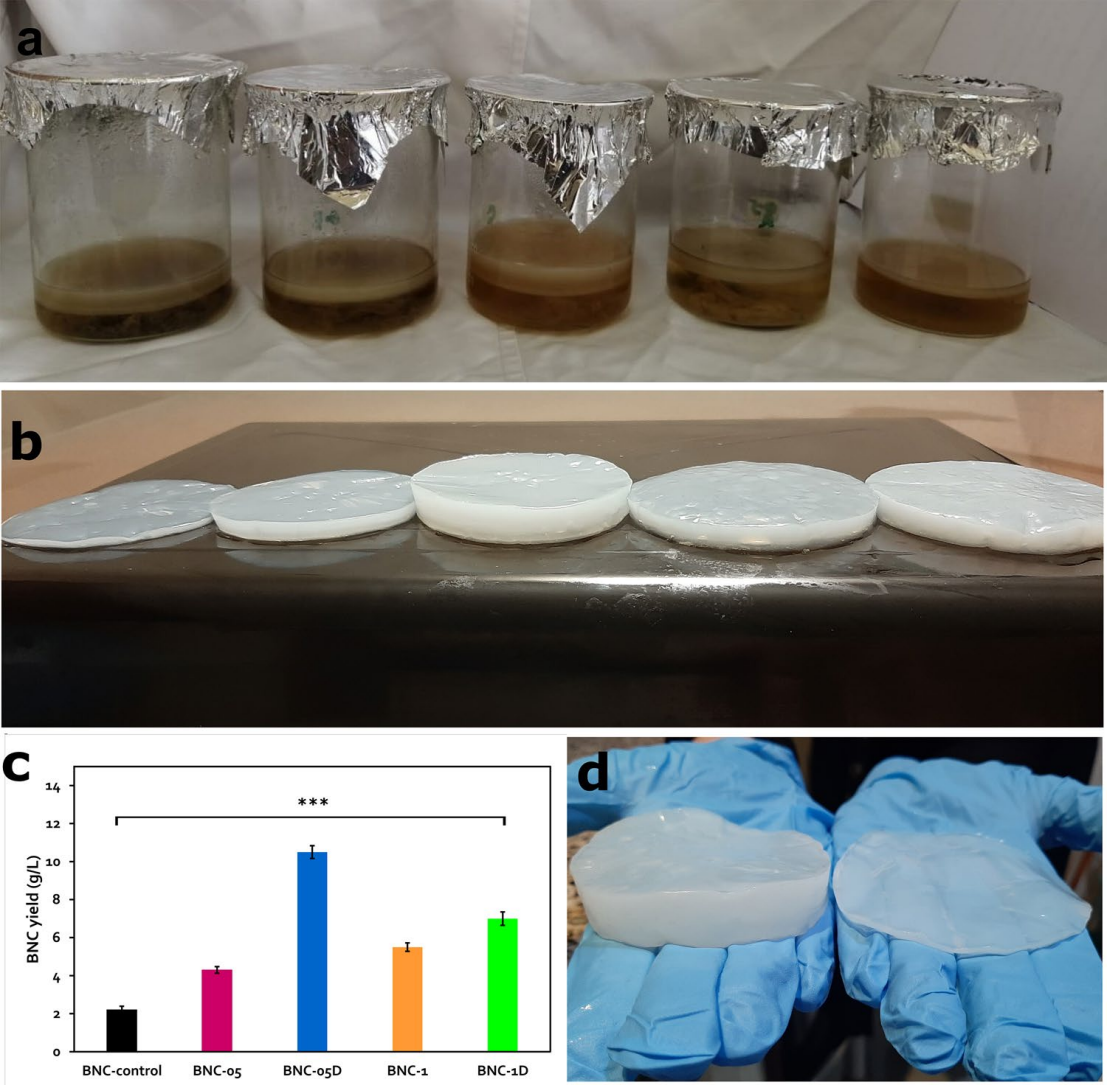
(**a**) Digital image for the propagation of the control and the irradiated cultures of the strain *K. hansenii* KO28, (**b**) the synthesized five BNC products after washing (from left: BNC-control, BNC-05, BNC-05D, BNC-1, and BNC-1D, (**c**) the BNC yield of the irradiated cultures of the strain *K. hansenii* KO28 comparing to that of the control culture. Values represent averages ± StD and ***P < 0.001 for the multiple comparison when n = 6, and (**d**) digital image reveals the BNC yield improvement of the BNC 05D (left) versus the ordinary production by the control culture.

Meanwhile, the doses 0.5, 0.5D, 1, and 1D kGy exerted distinctive enhancing influence on the BNC productivity (g/L), where the BNC yield escalated to reach 4.3, 10.5, 5.5, and 7 g/L, respectively, (Fig. 2c); versus only 2.2 g/L by the control culture. The digital images in Fig. 2 compare the cultures and the resulted BNC films of the control and irradiated bacterial cultures.

Cheng et al. reported that gamma ray-driven mutagenesis can trigger genetic or metabolic alterations in the treated cells, where the gamma ray interacts with the intracellular molecules, especially water molecules, emerging free radical flood. These plenty of free radicals strike the key functional genes and cause gene devastation and recombination^19^. We assumed that further examinations were necessary to configure the reasons beyond excessive BNC yield synthesized by the irradiated cultures.

### Time course of BNC production

We monitored the BNC production by the control and irradiated cultures of the bacterial strain *Komagataeibacter hansenii* KO28 throughout ten days, recording the BNC yield of each culture every 24 h.

**Figure 3:**
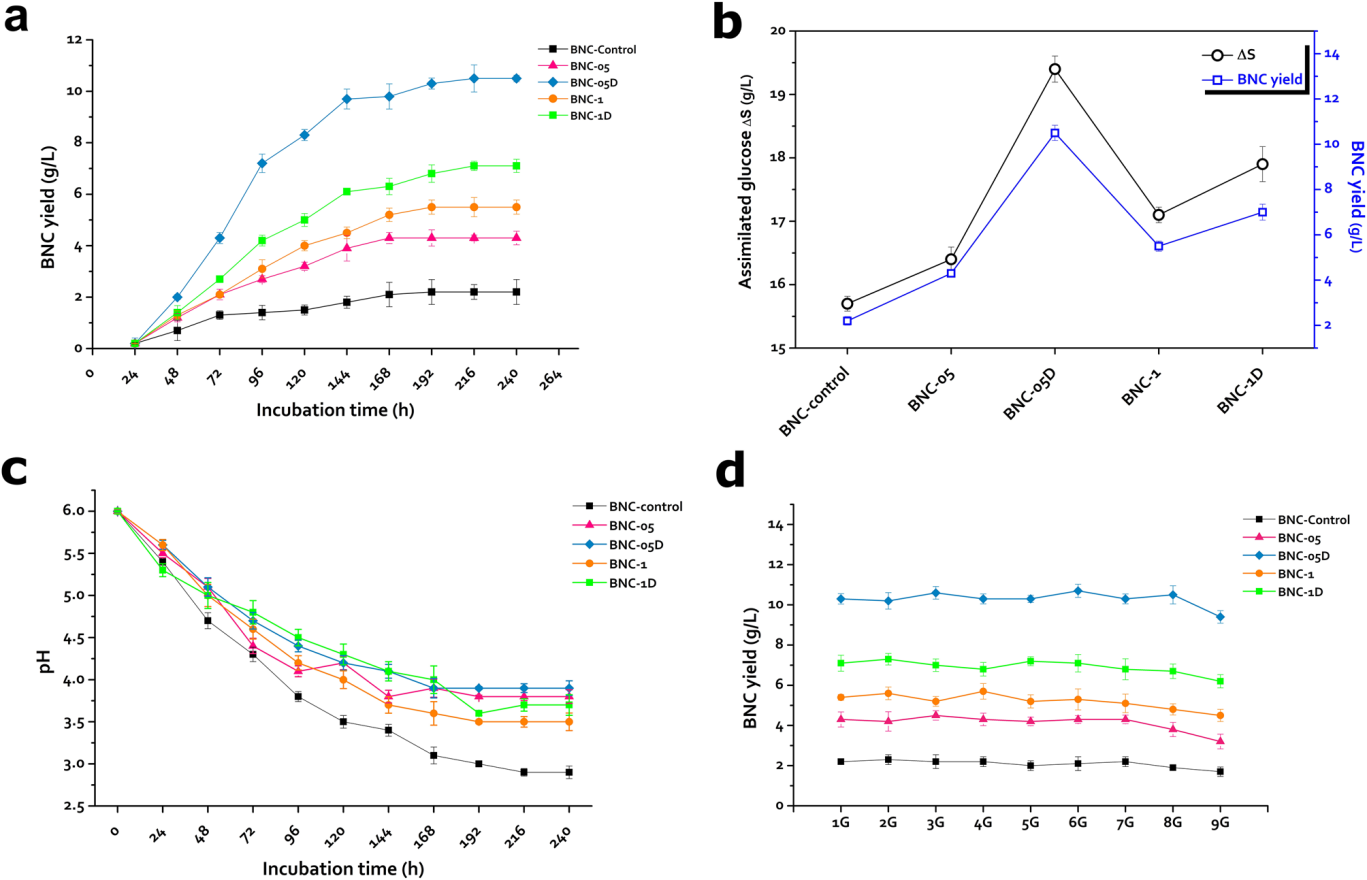
(**a**) The time course of the control and irradiated cultures of the strain *K. hansenii* KO28 throughout 240 h, (**b**) the glucose assimilation tendencies (ΔS) of the five cultures (g/L) versus the corresponding BNC yield (g/L), (**c**) pH trendlines of the five cultures through a time course of 240 h, and (**d**) the BNC yield synthesized by nine generations of the studied five cultures of the strain *K. hansenii* KO28.

The outcomes of this investigation indicated that the general BNC production rate is relatively following comparable tendency for both the control and the irradiated cultures (Fig. 3a). The primary BNC composition emerged after 24 h of starting incubation and kept rising until the 8th day and the 9th day for the control and irradiated cultures, respectively.

Remarkably, The BNC production exhibited by the culture treated by dual 0.5 kGy doses appeared in a uniquely climbing rate, where more than 92% of the whole BNC yield was generated in the first six days, prior the BNC production rate had slowed down in the next four days until the incubation terminated.

We supposed that the irradiation dose either enhanced the proliferation rate of the culture, and subsequently increase the producing units (i.e. number of cells), or immensely improves the efficiency of the singular bacterial cell to produce the BNC by polymerizing every available glucose residue into nanocellulose chains.

Regarding the hypothesis of the impact of irradiation scheme on the number of cells, there were no serious variation in the turbidity of the control and irradiated cultures or the shape of the produced films after ending the production time course. Moreover, the cells entrapped in the thick films of the irradiated cultures made the precise comparison of the CFU comparison for the five cultures quantitatively is impractical.

Alternatively, we speculated that concluding the assimilated glucose (ΔS) for the five cultures could be a good proof about the irradiation-relied metabolic modifications. (ΔS) value for any culture can be estimated from the difference between the medium glucose concentration at the beginning and end of the incubation period (g/L). Accounting the (ΔS) values for the five cultures revealed that the irradiated cultures exerted higher competency of glucose assimilation than the control culture in general. Furthermore, the proportions of the exploited glucose were fully relevant to that of the BNC yield for the five cultures (Fig. 3b).

Another clue could be deduced from the pH trend of the five cultures throughout the whole incubation time course. Figure 3c declares the major variation of the pH tendency of the control and irradiated cultures of the strain *K. hansenii* KO28 throughout 240 h of incubation. The pH of the control culture showed accelerated and constant declining trend continued to the end of the incubation period, reaching 2.9. Meanwhile, the irradiated cultures followed the same trend but with more flattened pH decrease, to record pH values ranged between 3.5 and 3.9.

Worthy to mention that the pH decrease associated with the cultivation of the genus *Komagataeibacter* is correlated with a distinctive metabolic feature of excessive gluconic acid synthesis once the cells begin to assimilate glucose residues, hence the culture pH turns more acidic. This metabolic activity is always corresponding with the bacterial propagation, and the consumed glucose units is certainly subtracted from the glucose capital which would be polymerized into BNC^11^, together with the impairing effect of the intense acidic environments on the BNC productivity^25^. Herein, the pH trendlines of the irradiated cultures in general were not compatible with the higher glucose assimilation rates exhibited in Fig. 3b. Therefore, we presumed that the gamma ray treatment in our case was capable of redirecting the metabolic system of the irradiated cultures, in varied extents, to maximize the glucose consumption for the BNC production pathway rather than increasing the multiplication rate, and gluconic acid generation consequently.

The hereditary stability of any microorganism is a critical issue considering their extraordinary rate of multiplication. It was broadly reported that the nanocellulose-producing genus *Komagataeibacter* may irreversibly discontinue the production process (termed Cel^-^ mutants) as a result of experiencing a physical or chemical unusual conditions such as vigorous agitation^8^.

Herein, we pursued the impact of the irradiation scheme on the potentiality of nanocellulose production of the strain *Komagataeibacter hansenii* KO28 for nine generations of the control and the four irradiated cultures, where the cultures undergone the irradiation treatment was designated (generation 0). Figure 3d reveals that all the examined bacterial cultures exhibited accountable steady BNC yield trendlines by their first seven generations, while the BNC productivity declined starting from the 8th generation. This impairment was affirmed by the lower BNC outcomes by the 9th generation of the control and irradiated cultures, suggesting that the applied irradiation doses had no serious influence on the hereditary stability of the studied cultures from one generation to another, where the subsequent generations of the four irradiated cultures exposed a BNC production tendency almost similar to that of the control culture.

According to the United States Pharmacopeia (USP) recommendations for the best microbiological procedures, the original strain should be preserved in multiple copies, where the number of sub-culturing should not exceed five times to avoid the spontaneous phenotypic alterations or mutations in the bacterial cells^26^. Therefore, we assume that the irradiated bacterial cultures will exhibit no susceptibility regarding the BNC productivity as long as such recommendations are appreciated.

### Microstructure by FESEM

The fibrous morphology of the BNC products of the control and the four irradiated cultures were compared by FESEM inspection.

**Figure 4:**
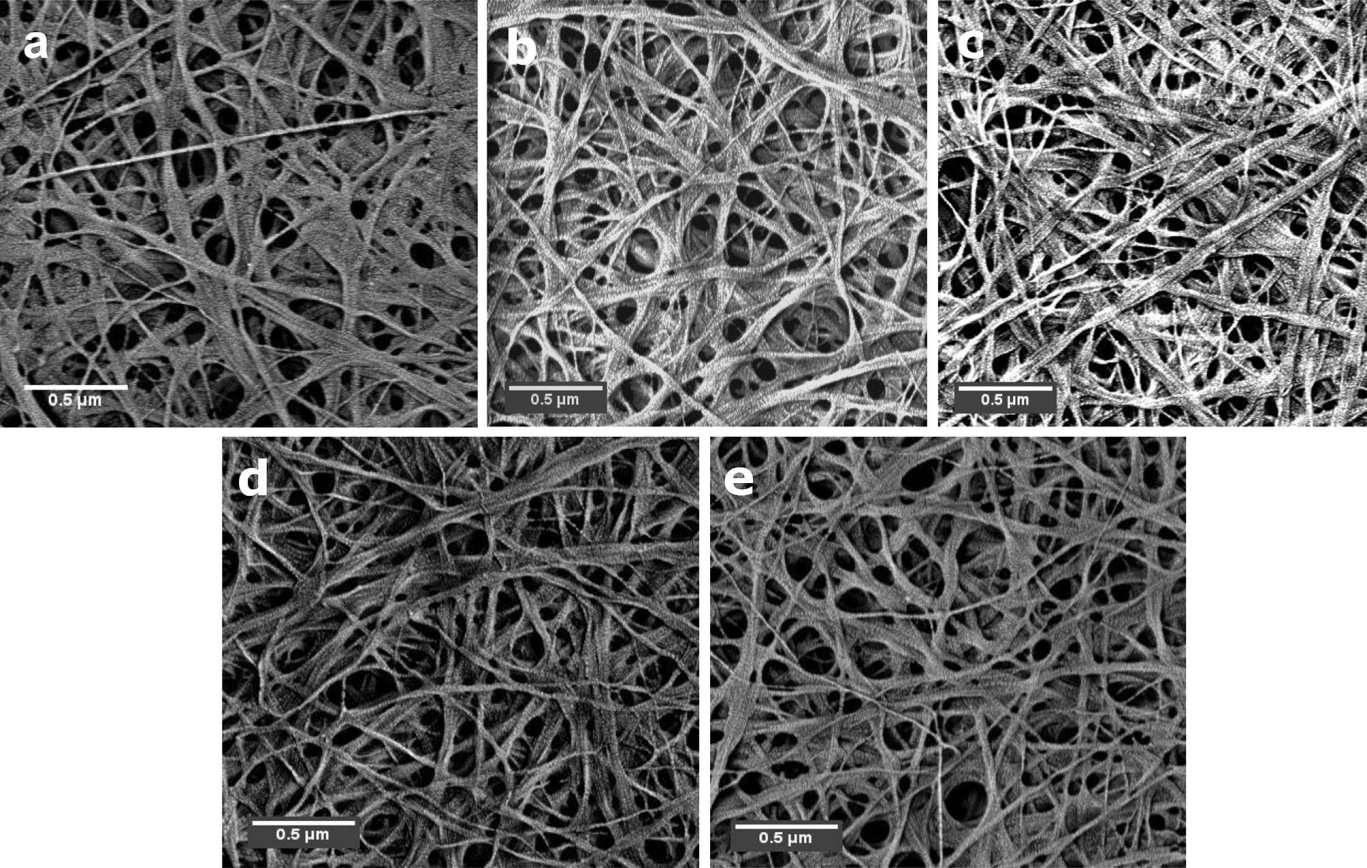
SEM of the (**a**) BNC-control, (**b**) BNC-05, (**c**) BNC-05D, (**d**) BNC-1, and (**e**) BNC-1D produced by the control and irradiated cultures of the strain *K. hansenii* KO28 (Scale bar: 0.5 µm).

The FESEM imagery in Fig. 4 shows a typical shape of the 3D nanofibrous web, where the thickness of the BNC nanofibers ranged between 5 and 85 nm. The examination of the five BNC specimens manifested no significant contradistinction between the control and irradiated BNC products from one side, or even among the four irradiated specimens regarding the fiber’s diameters, or the branching patterns.

Florea and his coworkers reported that the strain *Komagataeibacter hansenii* 53,582 regulates the BNC production via main four systems: *acs*A, B, C, and D, where *acs*A and *acs*B responsible for polymerizing UDP-glucose residues into cellulose chains. Meanwhile, the *acs*C is an outer membrane pore system acts for extruding the formed cellulose chains, where their crystallinity is regulated by the periplasmic system *acs*D^27^. Aside from influencing the BNC yield, we suppose that the irradiation doses had a mild or no impact on the *acs*C pore systems, where the fibers microstructure of the control and irradiated BNCs showed no considerable variations.

### Surface area investigation

The BET surface area of each BNC product was determined to inspect the influence of the culture irradiation on the corresponding microstructure of the specimen.

Herein, the examined BNC samples showed a variable values of specific surface area (Table 1), where the highest value was shown by the BNC-1, while the control BNC product came second, recording 62.3 and 54.4 m^2^/g, in respective order. Regarding the mean size of pores, the BNC-1 showed the largest pore size (27.2 nm), while the BNC-05D and BNC-1D came in the second and third extents with comparable values (22.16 and 21.3 nm, respectively). In terms of the pore volumes, the whole BNC specimens showed relevant values, except the BNC-1D and the BNC-control which recorded 0.29 and 0.26 cm^3^/g. However, the proportion of the nitrogen adsorption by each BNC sample is directly proportional to its surface area (m^2^/g). Ashrafi et al. elucidated how the gas adsorption–desorption inspects the operating pores, in addition to the closed channels and blind pores which have no actual association with the material permeability^28^.

**Table 1:** BET findings of the BNC artifacts of the control and irradiated cultures of the strain *K. hansenii* KO28.

	Specific surface area (m^2^/g)	Average pore diameter (nm)	Pore volume (cm^3^/g)
BNC-control	54.4	12.3	0.26
BNC-05	42.9	13.6	0.14
BNC-05D	50.03	22.16	0.19
BNC-1	62.3	27.2	0.11
BNC-1D	41.9	21.3	0.29

### Water retaining properties

The capability of the BNC to retain water has a substantial character for multiple applications such as; biomedical applications, food and food additives, adsorption, and not ending to the cosmetics and pharmaceutical emulsions^6^.

The water holding capacity (WHC) of the BNC is mainly owing to the multiplicity of the hydroxyl groups projected all through its adjacent glucan chains, which contribute with the elevated porosity and surface area per mass unit to endow the BNC with its prominent WHC^10^.

**Figure 5:**
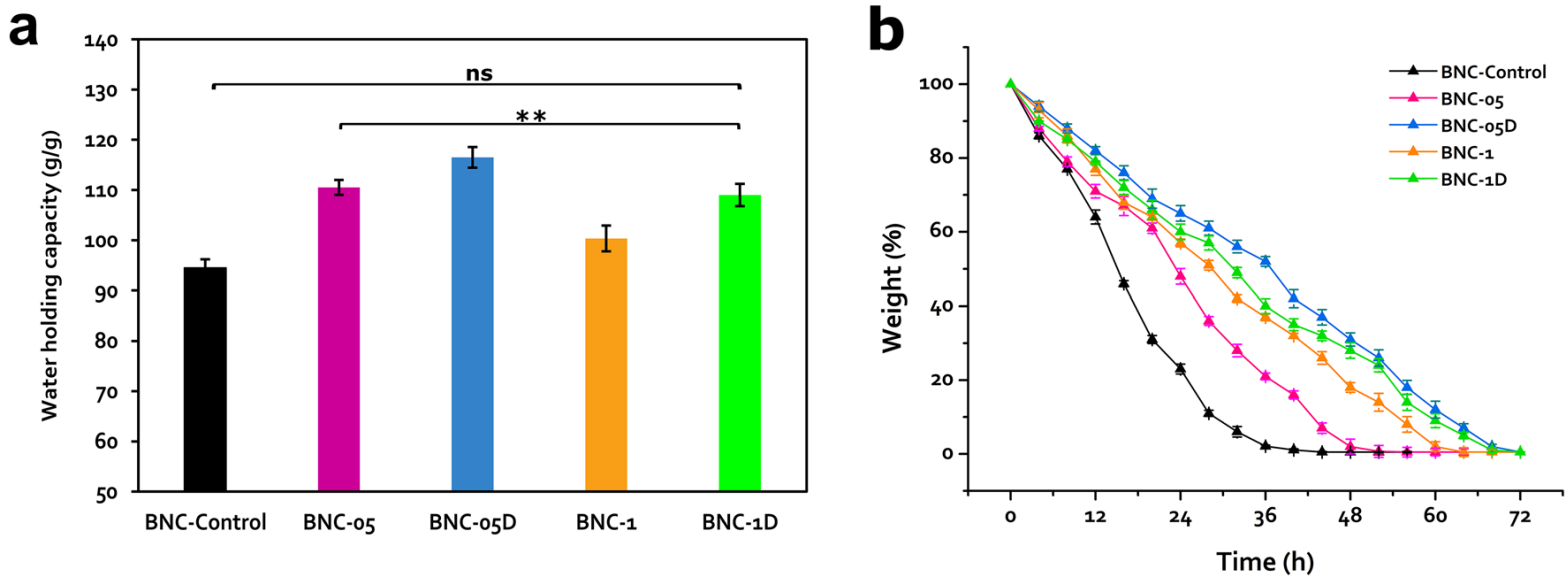
(**a**) water holding capacity (WHC), and (**b**) water release rates (WRR) of the control and irradiated cultures of the strain *K. hansenii* KO28. Values represent averages ± StD. where **P < 0.01, while (ns) refers to non-significant results where *P* > 0.05 for the multiple comparison when n = 6.

Here, the results manifested that the employed irradiation plan not only affected the BNC yield, but the ability of the BNC products to retain water as well. The BNC-05D exhibited the highest WHC improvement; recording about 116 g/g compared to the BNC generated by the control culture which showed only about 95 g/g (Fig. 5a). The BNC-05 and BNC-1D products came in the second and third ranks with comparable WHC values; 110 and 109 g/g, respectively. Finally, the BNC-1 film showed the lowest WHC improvement with only 100 g/g.

Referring to the water release rates (WRR) of the BNC products, it seems that the irradiation treatment had an impact on the potentiality of each BNC film to retain water, where all the four BNC products, comparing to the BNC-control, exerted higher invulnerability against dryness. Figure 5b depicts how the BNC-control film lost almost all its water content throughout only 40 h, while the BNC films produced by the irradiated cultures, particularly the BNC-05D, BNC-1, and BNC-1D, revealed prolonged water evaporation rates taking up to 72 h. At the test end, all the tested films exhibited fully dry texture, which was confirmed by constant weights even with the extended maintaining in air as a sign of the full excluding of moisture.

Rebelo and coworker reported the impact of the thickness and diameter of the BNC film on its dehydration time; postulating that the higher the thickness and/or diameter of the film, the slower the water evaporation rate^29^. Hence, in our investigation we examined films of the same diameter to avoid the resulting overlapping. Nevertheless, we predicate that the WHC and WRR of the BNCs were affected by the varied thickness of films.

Additively, the impact of the irradiation scheme probably extends to affect the crystalline and chemical composition (namely the density of the hydroxyl group). Therefore, we assumed that extra examinations of the physical and chemical features of the produced BNCs would be inevitable.

### Mechanical properties

We evaluated the stress–strain performance of the produced five BNC films to investigate the impact of the applied irradiation scheme on the mechanical properties of the produced BNC. Worthy to mention that the mechanical properties of the produced BNC are significant for a wide range of applications such as textile fabrication^30^, food packaging^31^, cementitious compositing^32^, and not ending with the biomedical applications^33^.

We summarized the values of tensile (MPa), elongation at break (%), and Young’s modulus for the five BNC products in Table 2. Regarding the tensile aspect, the BNC-05D film exhibited both the highest mean values of the tensile and Young’s modulus; recording an improvement of approximately 12 and 18%, respectively, if compared with the corresponding values of the BNC-control.

**Table 2:** Mechanical properties of the BNC films produced by the control and irradiated cultures of the strain *K. hansenii* KO28.

	Tensile strength (MPa)	Elongation at break (%)	Young’s modulus (MPa)
BNC-control	54 ± 4	3.7 ± 1.9	16 ± 2
BNC-05	49 ± 7	4.1 ± 2.6	14 ± 4
BNC-05D	61 ± 6	4.4 ± 2.4	19 ± 4
BNC-1	59 ± 5	4.7 ± 1.8	14 ± 5
BNC-1D	48 ± 6	3.9 ± 2.2	16 ± 4

The BNC-1 attained the second highest tensile improvement comparing to the BNC-control, while its ductility was superior out of the whole tested films; representing about 1% improvement in the elongation at break comparing to the BNC-control.

Cellulose polymer is composed of self-assembled chains of β-1,4-linked anhydrous D-glucose units where the intermolecular hydrogen bonding and the Van der Waals forces promote the stacking of the adjacent chains to nanofibrils, which are further gathered to form larger fibrils. Furthermore, the molecular structure of these fibrils varies between crystalline and amorphous regions, where many factors govern which type is dominant and the proportion of each one. This unique crystalline structure is the main character which confers on the cellulose nanofibers some of their unparalleled properties such as the mechanical properties^34^. Molnàr et al. studied in detail the correlation between the crystalline structure and the mechanical behavior of the cellulose nanofibers^35^.

Martin-Martinez explained how the understanding of the behavior of the cellulose crystals is significant for designing up-to-demand nanocellulose products through a “bottom-up” process. Subsequently, this will make the manipulation of the nanocelluloses more facile and provide broader application fields^36^.

### X-ray diffraction

We accomplished a broad study of the diffractometry of the BNC produced by the control and irradiated cultures of the strain *K. hansenii* KO28 to scrutinize the impact of the applied irradiation doses on the crystalline structure of the BNC artifacts, in terms of the crystallite size (CrS), inter-planar distances (*d*-spacing), and crystallinity index (CrI %).

**Figure 6:**
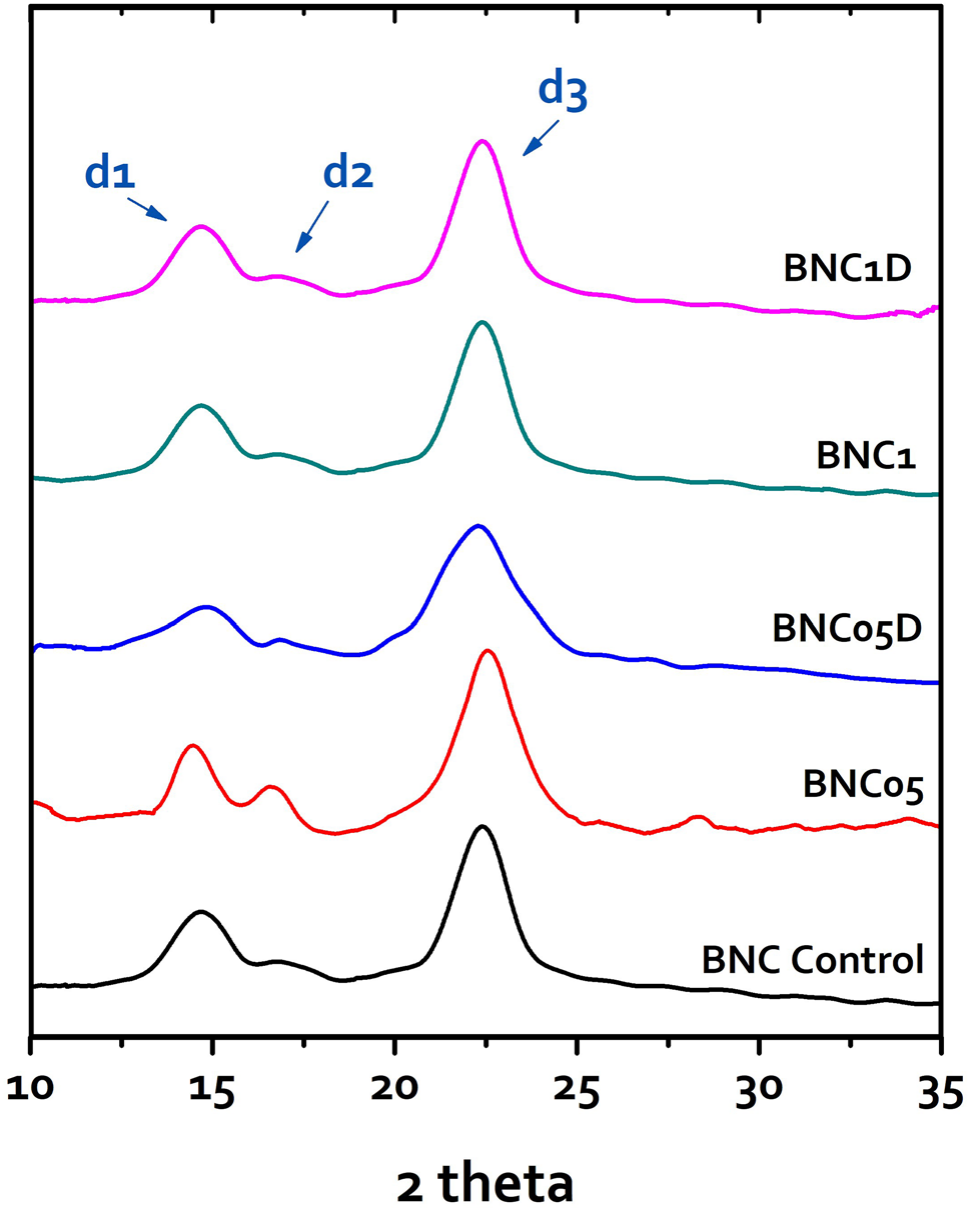
X-ray diffraction profiles for the BNC artifacts produced by the strain *K. hansenii* KO28, where the three main peaks are represented as *d*1, *d*2, and *d*3.

Figure 6 represents a comparison of the five X-ray diffraction portraits, where they showed up in the characteristic diffraction profile of cellulose I, including three main peaks; d1, d2, and d3.

The peak d1 appeared at 2θ = 14.66° ± 0.3°, as a mean value of the peaks of the BNC products, and ascribed to (100) plane of I_α_ or to the plane (1–10) of I_β_. The peak d2 spiked at 2θ = 16.7° ± 0.2°, and accredited to (010) plane of I_α_ or (110) plane of I_β_. The d3 at 2θ = 22.4° ± 0.1°, and could be a contribution of (110) plane of I_α_, the (200) plane of cellulose I_β_.

Moreover, the percentage of the crystalline allomorph in the produced five BNCs appeared to be dissimilarly influenced by the irradiation treatment. Whilst, the BNC-control comprised about 74%, the doses of 0.5 kGy and dual 1 kGy relatively had a mild impact on the percent of the crystalline allomorph; recording about ± 1% change.

On the other hand, the doses of dual 0.5 and 1 kGy appeared to exhibit a distinctive effect on the crystalline proportion in the produced BNC, where their products’ crystallinity attained about 80% and 77%, respectively.

The quantitative estimation of the crystallite sizes (CrS) and the *d*-spacing (d) for each peaking lattice are enumerated in Table 3. Out of the inspected five BNC products, the irradiation dose 0.5 kGy appeared to have a dramatic impact on the crystallite size. When the culture treated with a single 0.5 kGy dose, the BNC exhibited the highest crystallite sizes in the planes (100) and (1–10), while treating the culture with a dual 0.5 kGy dose plummeted the crystallite size to record the lowest values out of the whole five products.

**Table 3:** A brief of the crystalline properties; *d*-spacing (*d*), crystalline size (CrS), and crystallinity index (CrI) of the BNCs produced by the control and the irradiated cultures of the strain *K. hansenii* KO28.

	d1			d2			d3			CrI (%)
	2 theta	*d* (nm)	CrS (nm)	2 theta	*d* (nm)	CrS (nm)	2 theta	*d* (nm)	CrS (nm)	
BNC-control	14.66	0.6	6.2	16.76	0.52	6	22.4	0.39	7.4	74
BNC-05	14.46	0.61	8.5	16.58	0.52	9.1	22.56	0.3	6.9	73
BNC-05D	14.82	0.59	3.3	16.84	0.53	5.7	22.3	0.35	4.3	80
BNC-1	14.7	0.62	6.2	16.75	0.52	6	22.4	0.39	7.4	77
BNC-1D	14.68	0.61	6.2	16.79	0.53	6.1	22.4	0.39	7.3	75

This obviously proposed that applying the second irradiation 0.5 kGy dose had a distinctive impact on the periplasmic *acs*D system commanding the cellulose chain crystallization, resulting in relatively tinier crystallites. However, the case was dissimilar to irradiation by the 1 kGy or the dual 1 kGy doses, suggesting that the irradiation doses > 0.5 kGy promoted some amendments in the cellular metabolism or the BNC yield, but it does not extend to the crystalline structure such as the crystallite size or the *d*-spacing.

### Thermogravimetric analysis

The outcomes of the thermogravimetric analysis showed that the five BNC products exhibited almost the same decomposition pattern throughout the scanned temperature range (Fig. S2).

**Figure 7:**
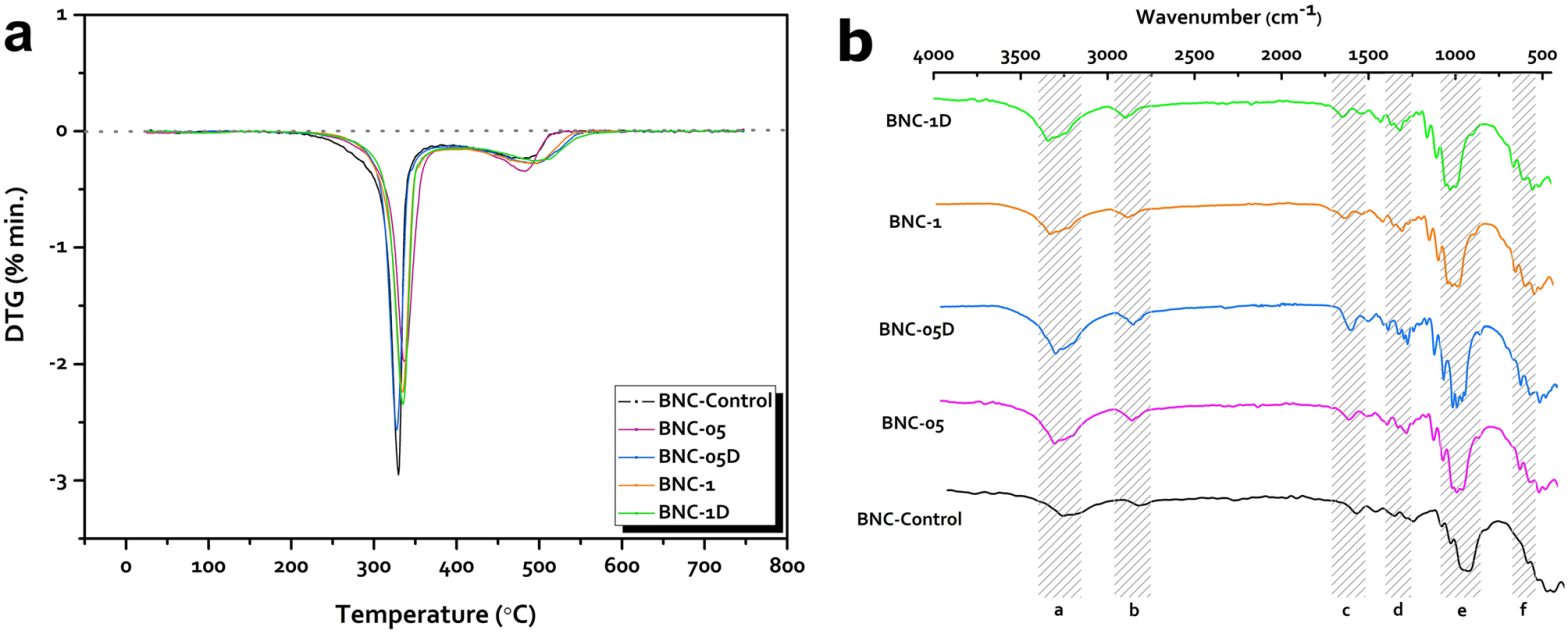
(**a**) The derivative thermogravimetric patterns (DTG) of the BNC products of the control and irradiated cultures of the strain *K. hansenii* KO28, and (**b**) FTIR spectra of the BNC samples produced by the five cultures, where the letters (**a**–**f**) is corresponding to the most distinctive peaks and the relevant assignments are listed in Table 5.

The derivative thermogravimetry (DTG), plotted in Fig. 7a and listed in Table 4, declares that the decay temperature degrees for the produced BNCs ranged from 328 to 336 ℃. Comparing to BNC-control, it can be concluded that the applied irradiation scheme for the bacterial culture had a mild or no impact on the BNC structural bonds responsible for fastening the cellulose nanofibers and microfibers to each other, keeping them firmly attached to grant the BNC its extraordinary mechanical properties.

**Table 4:** A summary of the thermogravimetric tendencies of the BNC samples synthesized by the control and irradiated cultures of the strain *K. hansenii* KO28.

	Mass loss (%)			
	25–250 ℃	251–450 ℃	451–650 ℃	DTG
BNC-control	3.3	85.85	98.2	329
BNC-05	3.83	82.91	98.61	336
BNC-05D	3.34	79.33	98.42	328
BNC-1	3.2	80.7	98.9	333
BNC-1D	3.55	79.8	99.07	335

### Fourier transform infra-red analysis

The results of the IR investigation for the five BNC products were depicted in Fig. 7b, and the band assignments were listed in Table 5^10^. Moreover, the two bands emerged at 1540 and 1640 cm^-1^ for the five examined samples are assigned to the amide bond, which are most probably correlated to the protein remnants and cellular debris which kept attached to the BNC samples even after the washing steps. However, we assume that it is anticipated considering the elevated thickness of the BNC films produced by the irradiated cultures.

**Table 5:** FTIR band assignments of the BNC produced by the control and irradiated cultures of the strain *K. hansenii* KO28. The bands listed with the corresponding legends on Fig. 7b.

Legend on the Fig. 7b	Absorption band (cm^-1^)	Band assignment
a	3340 ± 5	O–H stretching
b	2892 ± 5	C–H stretching
c	1645 ± 5	water bending vibrations
d	1376 ± 1	C–H bending
e	1002–1029	C–O at C3; C–C stretching; and C–O at C6
f	662 ± 5	C–O–H out of plan bending

The IR profiles of the five BNC products were correlated with cellulose I, without clear evidence for existence of cellulose II. Moreover, the bands manifested at 3240 and 3270 cm^-1^ affirm the existence of both I_α_ and I_β_ allomorphs in the tested BNC. The crystalline I_α_ fraction (F_α_^IR^) was estimated for the five BNC products Table 6 as an attempt to configure the influence of the irradiation application on the crystalline properties of the BNC products. It is clearly evident that the I_α_ amount in the produced BNC samples did not greatly affected by the applied irradiation scheme to the bacterial cultures.

**Table 6:** List of the I_α_ proportions in the five produced BNC samples.

	A_750_	A_710_	*F*_*α*_^*IR*^
BNC-Control	0.94133	0.92164	0.86
BNC-05	0.88359	0.86052	0.86
BNC-05D	0.73684	0.65994	0.87
BNC-1	0.82886	0.78699	0.86
BNC-1D	0.88359	0.86052	0.86

Interestingly, the band at 3340 cm^-1^, assigned for the hydroxyl group, proved higher hydroxyl group density for the irradiated samples rather than that of the control. These outcomes are consistent with the previously revealed WHC results, explaining how the BNC-control possesses inferior capability of water retaining, out of the five studied BNC products.

We presume that this data can stand beside those concluded from the XRD to give a clear figure for the structural characters of the produced BNCs.

Ultimately, we believe that the outcomes of our research are a massive leap forward in defining the best technique for feasible BNC mass production. Examining the capability of the irradiated strain to produce high yielded BNC via assimilating low-cost substrates, as well as revealing the impact of irradiation dosages on BNC yield and characteristics through a genetic perspective are listed on our future workplan.

## Conclusion

We identified a novel BNC-producing strain as *Komagataeibacter hansenii* KO28. the isolated strain was treated with multiple doses of gamma ray (0.5, 1, 2, 3 singular and double doses) and their impact on the BNC productivities was evaluated. The four cultures encountered positive BNC yield impact (treated with 0.5, dual 0.5, 1, and dual 1 kGy) were compared with the wild-type (control) culture in regards of the BNC yield and hereditary stability, and BNC physical properties. FESEM revealed no significant difference in terms of fibers diameter or outspreading pattern, while a moderate variation exposed in the surface area values. Out the of the examined five BNC artifacts, the BNC-1 showed the highest surface area and ductility, while the BNC-05D exerted the best water holding capacity, best water retention, highest tensile strength, and highest Young’s modulus as well. The thermo-decay profiles of the examined BNCs were comparatively relevant, but the crystalline properties varied, suggesting another influence of the double irradiation doses on the cellular crystallization systems. Eventually, the BNC products showed comparable IR spectra, with more dense hydroxyl peaks exhibited by the BNC films came from irradiated cultures.

## Materials and methods

### Materials

D-Glucose and citric acid were purchased from Fisher chemical; peptone, yeast extract and agar were Conda Lab supplies; and the sodium di-basic hydrogen phosphate was provided from Sigma Aldrich and used as received.

### Isolation and identification

The BNC was produced by the strain *Komagataeibacter hansenii* KO28, which was isolated from a flower garden in (SRTA-City) campus. For identifying the obtained bacterial isolate, we accomplished a sequential scheme of morphological and physiological examinations, prior to the partial sequencing of the 16 s rRNA gene according to Sanger et al.^24^.

Eventually, we employed the Basic Local Alignment Search Tool (BLAST) to compare the obtained sequences with those of the correlated strains on the National Center for Biotechnology Information GenBank (NCBI GenBank) databases, while the Neighbor-Joining tree for the identified strain was plotted via MEGA software (V6) through the bootstrap analysis with a value of 500.

### Scheme of culture irradiation

The treatment of the strain *Komagataeibacter hansenii* KO28 by gamma irradiation was carried out adopting ^60^Co gamma rays at a dose rate of 0.77 kGy/h^17^. Briefly, aliquots of 10 ml volume of the bacterial culture were prepared, then, divided into three sets; Control, set (A), and set (B).

The control set represented the wild bacterial culture which was not subjected for any irradiation treatment. Set (A) included the cultures treated with single irradiation doses viz. 0.5, 1, 2, and 3 kGy. Eventually, set (B) which underwent the irradiation treatment as same as set (A), then left to grow for 7 days and sub-cultured twice in fresh (HS) medium for 7 days each time to obtain the third generation, which re-irradiated with the corresponding radiation doses.

All in all, and throughout this scheme, we attained three culture sets; non-irradiated culture (control), single irradiated set cultures (4 doses), and dual irradiated set cultures (4 doses).

The BNC products were designated with the irradiation dose received by the generating culture, with addition of “D” letter to the BNC products came from the cultures treated with dual doses.

### Time course of the BNC production

All the following trials were carried out in six replicates, where we estimated their averages and standard deviations.

The examined cultures were grown in beakers of 5oo ml capacity included 100 ml of sterile (HS) medium. The inocula represented 10% of the final volume, and the initial pH was adjusted at 6 using 0.1 N H_2_SO_4_. The inoculated beakers were sealed and moved to the incubator at 30 ℃ for 10 days statically. At the end of the incubation session, the BNC films were collected and washed twice in boiling distilled water, and twice in 0.1 N NaOH solution, 15 min each time, prior to applying distilled water many times until the neutral pH of the BNC films was restored. The neutral films were maintained in distilled water in 4 ℃ until further examination. The medium remnants were assessed for the glucose concentration and pH.

For determining the BNC productivity of each culture, we collect the wet BNC films of each culture and extended them on PTFE plates where they moved to oven dryer (Carbolite PF120-England) of 60 ℃ for 6 h, then we weight each film adopting high precision balance (Sartorius CP224S-Germany). The yield of each culture was recorded (g/L) and compared versus that of the control culture to evaluate the irradiation impact on the BNC productivity.

### Glucose determination

The concentration of glucose of the remnants of the production media were assessed via by the sulfuric acid/anthrone colorimetric method^37^.

### FESEM imaging

The BNC films were investigated by field emission scanning electron microscope (FESEM) by Quanta™ Field emission gun (FEG 250) at a voltage of 5 kV after sputtering samples with gold nanoparticles for 2 min.

### BET investigation

Brunauer, Emmett, Teller (BET) surface area, pore diameter, and pore volume of each BNC product were investigated. Prior to inspection, each BNC sample underwent a freeze-drying procedure, then degassed at 150 °C under reduced pressure. The investigation was accomplished employing (BELSORP-miniX) instrument.

### Water holding capacity (WHC) and water release rate (WRR)

Inspection of the impact of the irradiation scheme on the water holding capacity (WHC) of the produced BNCs was done by collecting never dried films and the excess water was wiped out by a paper towel. The samples were weighted (W_wet_), and then left to dry in room temperature for 72 h.

Afterwards, the films were dried at 40 °C for 12 h to evaporate any humidity content, before recording the final weight of each film (W_dry_). The water holding capacity for each BNC sample can be concluded from the following equation:

1 $${\text{WHC }}\left( {{\text{g}}/{\text{g}}} \right) \, = {\text{ W}}_{{{\text{wet}}}} \left( {\text{g}} \right)/{\text{W}}_{{{\text{dry}}}} \left( {\text{g}} \right)$$

Regarding the water release rate (WRR), never dried circular BNC films, 5 cm diameter, were weighted (W_wet_). The thickness of the wet films; BNC-control, BNC-05, BNC-05D, BNC-1, and BNC-1D were determined to be 3, 6, 13, 8, and 9 mm, in respective order. Afterwards, the films were left on PTFE plates in room temperature with frequent weight recording every 4 h. The water release behavior was plotted as weight percentages of the (W_wet_) versus time (h).

### Evaluation of the mechanical properties

The mechanical properties of the BNC films were inspected by a universal testing machine (AG-1S, SHIMADZU, Japan). A rectangle of 2 × 5 cm was cut from each sample and we assessed the thickness using (Sealey AK9635D) micrometer. To provide a constant strain rate on measurement, the cell preload set to 5 N and testing speed of 10 mm/min was applied.

### Analysis of the X-ray diffraction

X-ray diffraction of the BNC produced by the control and the irradiated cultures was configured using (labX XRD-6100, Shimadzu, Japan). The utilized CuKα radiation wavelength was (λ = 1.54 Å), generated at a voltage of 40 kV and a filament emission of 30 mA. BNC samples were scanned at 2θ range of 5°– 60° at a scan speed of 0.5° min^−1^.

The investigation outcomes for each BNC sample were plotted through Origin software 8.0, and the Crystallinity was estimated through the equation:

2 $${\text{CrI }}\left( \% \right) \, = \, \left[ {{\text{I}}_{{({2}00)}} - {\text{ I}}_{{({\text{am}})}} /{\text{I}}_{{({2}00)}} } \right] * {1}00$$

where I_(200)_ is the highest value of the peak at 2θ = 22°, I_(am)_ is the minimum value between the peaks 2θ = 22° and 2θ = 16°.

The crystallite size was concluded from Scherrer’s equation:

3 $${\text{CrS }} = {\text{ k}}\lambda /\left( {\beta {\text{ cos}}\theta } \right)$$

where (k) is the dimensionless Scherrer constant for cubic crystals = 0.9, (λ) is the X-ray wavelength, (β) is the peak full width at half maximum (FWHM), and (θ) is the diffraction angle.

Bragg’s equation was used to determine the atomic inter-planar spacing (d) as following:

4 $${\text{d }} = {\text{ n}}\lambda /{\text{2 sin}}\theta$$

where (n) is the order of the peak plane.

### Thermogravimetric analysis (TGA)

Thermogravimetric analysis was performed using a TGA–DSC/DTA analyzer (Discovery SDT 650, TA instruments, USA) in air at a temperature ranged from room temperature to 750 ℃ and 10 ℃ min^-1^ heating escalation. The starting weight of the samples BNC-Control, BNC-05, BNC-05D, BNC-1, BNC-1D were 4.2, 3.8, 4.1, 3.6, and 4.09, respectively.

### Fourier transform infrared (FTIR) spectrophotometry

The five BNC products were examined by FTIR (FTIR-8400 S, Shimadzu, Japan) to investigate the footprints of the irradiation doses on the chemical composition of the nanocellulose comparing to the control one. The test was accomplished at spectra range 4000–400 cm^−1^ with 2 cm^−1^ resolution for 16 scans per measurement. According to Khajavi et al.^38^, the proportion of the allomorph Iα can be concluded from the equation:

5 $$F_{\alpha }^{IR} = \frac{{A_{750} }}{{A_{750} + kA_{710} }}$$

where “A_750_” and “A_710_” are the absorbance values at the corresponding wavenumbers, while “k” is a constant (k = 0.16).

### Statistical analysis

All the production trials were performed in six replicates, and the outcomes were statistically analyzed using GraphPad Prism software (Version 7). The data were analyzed employing one-way and two-way analyses of variance (ANOVA) with the Tukey’s test (honestly significant difference) for multiple comparisons. The values were expressed by averages ± SD, and the level of significance was at *p* < 0.05, where n = 6^39^.

## Supplementary Information

Supplementary Information.

## Data Availability

The partial sequence of the 16s rRNA gene of the adopted strain is available in the National Center for Biotechnology Information (NCBI) GenBank repository as Komagataeibacter hansenii KO28 and accession number MW819862 https://www.ncbi.nlm.nih.gov/nuccore/MW819862.
