# Supplementary material for: A turning point in the bacterial nanocellulose production employing low doses of gamma radiation
Source: Sci Rep. 2022 Apr 29;12:7012. doi: 10.1038/s41598-022-11010-4 (PMC9054840; doi:10.1038/s41598-022-11010-4)
Supplement: Supplementary file 1 — Supplementary Information. [file 41598_2022_11010_MOESM1_ESM.pdf]

Supplementary information

A turning point in the bacterial nanocellulose production employing low doses of gamma radiation

Ola E. A. Al-Hagar[1], Deyaa Abol-Fotouh[2*]

[1] Plant research department, Nuclear Research Center, Egyptian Atomic Energy Authority, 13759, Cairo, Egypt

[2] Department of Electronic Materials Researches, Advanced Technology and New Materials Research Institute (ATNMRI), City of Scientific Research and Technological Applications (SRTA-City), New Borg El-Arab City, 21934, Alexandria, Egypt

***Corresponding author**

dabolfotouh@srtacity.sci.eg
dabolfotouh@gmail.com

Tel: +2034593414

Results of the isolation and identification of the BNC-producing isolate

The isolate (KO28) was isolated from the soil of a flower garden. Once it was purified and grown on Hestrin-Schramm (HS) medium for 10 days, the characteristic floating pellicle of a hydrogel has showed up on the medium surface.

With the help of a plan of morphological and physiological examinations to determine its phenotypical features (table S1), the partial sequencing of the 16s rRNA gene indicated that the isolate (KO28) identified as the strain *Komagataeibacter hansenii*.

The strain data was deposited in the National Center for Biotechnology Information (NCBI) GenBank under as *Komagataeibacter hansenii* KO28 and accession number MW819862. Moreover, fig. S1 reveals the phylogenetic tree of the 16S rRNA including the position of *Komagataeibacter hansenii* KO28 compared to the correlated sequences on the NCBI GenBank database.

**Characteristics**	**Isolate (KO28)**
Gram stain	Gram -ve
Cell shape	Rod shaped
Motility	No
Colony color	Beige, opaque
Colony shape	Circular
Colony margin	Entire
Colony elevation	Convex
Growth anaerobic	-ve
Growth without acetic acid	+ve
Growth on	
Glutamate agar	+ve
Mannitol agar	+ve
Production of water-soluble brown pigment	-ve
Production of acids from:	
D-glucose	+ve
D-mannitol	-ve
D-sorbitol	-ve
Glycerol	+ve
D-fructose	-ve
Ethanol	+ve
Maltose	-ve
Sucrose	-ve
Cellulose production	+ve

Table S1. Morphological and physiological characteristics of the bacterial isolate KO28.

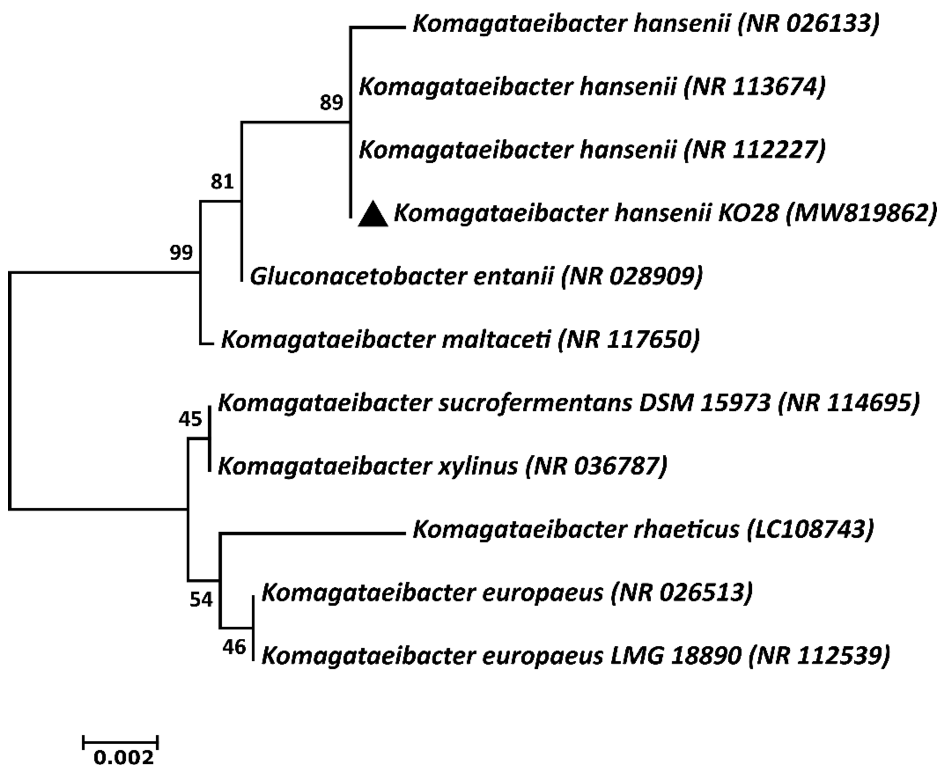

Figure S1. Phylogenetic tree of *Komagataeibacter hansenii* KO28 (▲) revealing its location to the most relevant bacterial strains relying on the 16S rRNA gene nucleotide sequences. The numbers corresponding to each strain represent the accession numbers of each 16S rRNA nucleotide sequences deposited in GenBank database.

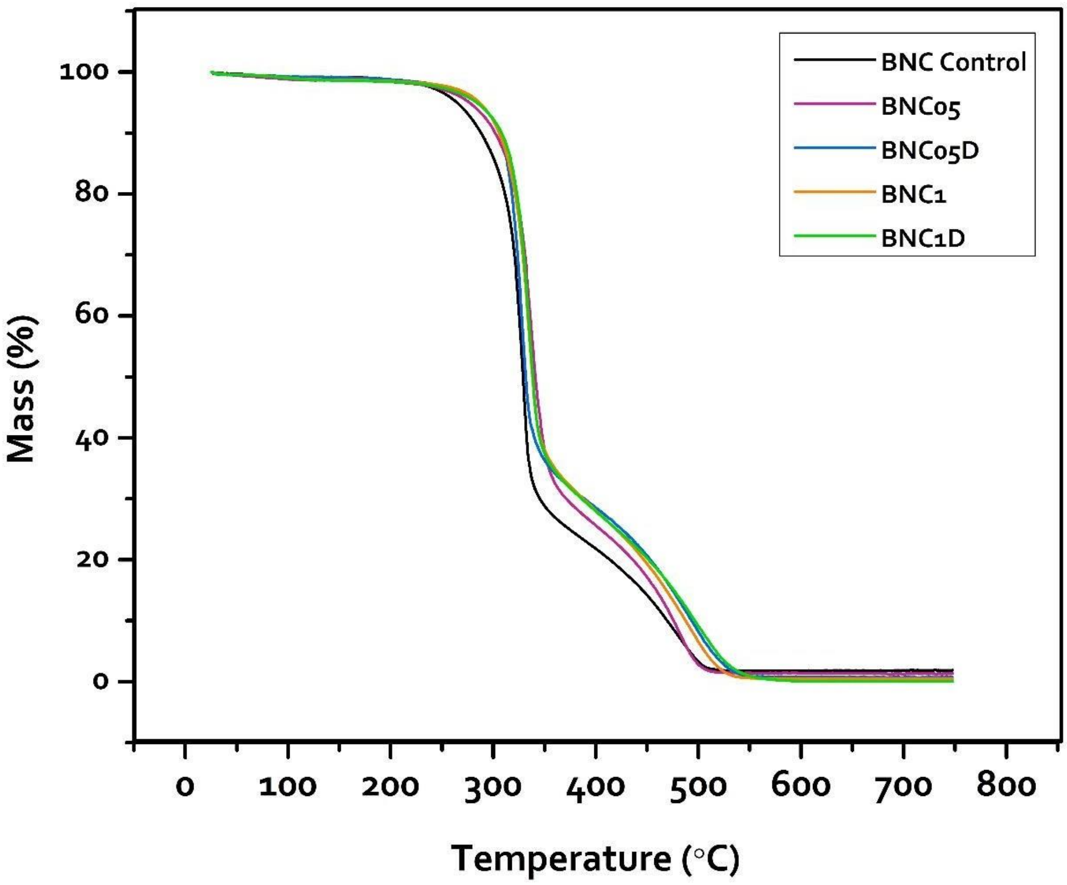

Figure S2. Thermogravimetry of the BNC produced by the control and irradiated cultures of the strain *K. hansenii* KO28.
